# Association of cytosolic sialidase Neu2 with plasma membrane enhances Fas-mediated apoptosis by impairing PI3K-Akt/mTOR-mediated pathway in pancreatic cancer cells

**DOI:** 10.1038/s41419-017-0191-4

**Published:** 2018-02-12

**Authors:** Shalini Nath, Chhabinath Mandal, Uttara Chatterjee, Chitra Mandal

**Affiliations:** 1 Cancer Biology and Inflammatory Disorder Division, Council of Scientific and Industrial Research (CSIR), Indian Institute of Chemical Biology (IICB), 4, Raja S.C. Mullick Road, Jadavpur, Kolkata, 700032 West Bengal India; 2 National Institute of Pharmaceutical Education and Research, IICB, CSIR, Kolkata, 700032 West Bengal India; 3 Department of Pathology, Institute of Postgraduate Medical Education and Research, Institute of Post-Graduate Medical Education and Research Hospital, Kolkata, 700020 West Bengal India

## Abstract

Modulation of sialylation by sialyltransferases and sialidases plays essential role in carcinogenesis. There are few reports on sialyltransferase, however, the contribution of cytosolic sialidase (Neu2) remains unexplored in pancreatic ductal adenocarcinoma (PDAC). We observed lower expression of Neu2 in different PDAC cells, patient tissues, and a significant strong association with clinicopathological characteristics. Neu2 overexpression guided drug-resistant MIAPaCa2 and AsPC1 cells toward apoptosis as evidenced by decreased Bcl2/Bax ratio, activation of caspase-3/caspase-6/caspase-8, PARP reduction, reduced CDK2/CDK4/CDK6, and cyclin-B1/cyclin-E with unaffected caspase-9. Neu2-overexpressed cells exhibited higher expression of Fas/CD95-death receptor, FasL, FADD, and Bid cleavage confirming extrinsic pathway-mediated apoptosis. α2,6-linked sialylation of Fas helps cancer cells to survive, which is a substrate for Neu2. Therefore, their removal should enhance Fas-mediated apoptosis. Neu2-overexpressed cells indeed showed increased enzyme activity even on membrane. Interestingly, this membrane-bound Neu2 exhibited enhanced association with Fas causing its desialylation and activation as corroborated by decreased association of Fas with α2,6-sialic acid-binding lectin. Additionally, enhanced cytosolic Neu2 inhibited the expression of several growth factor-mediated signaling molecules involved in PI3K/Akt–mTOR pathway probably through desialylation which in turn also causes Fas activation. Furthermore, Neu2-overexpressed cells exhibited reduced cell migration, invasion with decreased VEGF, VEGFR, and MMP9 levels. To the best of our knowledge, this is the first report of cytosolic Neu2 on membrane, its association with Fas, enhanced desialylation, activation, and Fas-mediated apoptosis. Taken together, our study ascertains a novel concept by which the function of Fas/CD95 could be modulated indicating a critical role of upstream Neu2 as a promising target for inducing apoptosis in pancreatic cancer.

## Introduction

More than 90% of pancreatic cancers are pancreatic ductal adenocarcinoma (PDAC), is fatal due to poor diagnosis and prognosis^1,2^. Because of its rapid progression, invasiveness, and drug resistance, most have metastatic cancers^3–6^. The multifaceted biological mechanisms remain mostly unknown.

Abnormal glycosylation and fucosylation are common features in cancers^7–11^. Hence these alterations play a significant role in modulating differentiation, signaling, adhesion, invasiveness, metastasis, and apoptosis^12^. Pancreatic cancer cells exhibited higher α2,3- and α2,6-linked sialic acids (SAs) which mainly affects its higher rate of metastasis^13,14^.

Enhanced SAs depend on the balance of SA-modulatory enzymes sialyltransferases and sialidases^15–17^. Elevated levels of the sialyltransferases are common in cancers including PDAC^18–25^. Mammalian cells have four sialidases namely lysosomal (Neu1), cytosolic (Neu2), membrane bound (Neu3), and luminal (Neu4) differing in their enzymatic property and substrate specificity. They are important for the balance of sialylation and behave differently during carcinogenesis^26,27^.

Neu2 expression is either very low or undetectable in normal human tissue with the exception of prostate cancer and myoblast^28–30^. Neu2 is repressed in leukemia, melanoma, and colon adenocarcinoma^31–33^.

Death receptor Fas (CD95) stimulates several signaling cascades for inducing apoptosis. This is commonly disrupted and implicated in tumor cell survival^34,35^. Both *O*- and *N*-linked glycans and α2,6-sialylations of Fas are reported in colon cancer^36^. The substrate specificity of Neu2 is toward glycoproteins/glycolipids and oligosaccharides. However, the functional significance of Neu2 in Fas-mediated apoptosis has received least attention.

PI3K–Akt pathway is upregulated in many cancers^37,38^. Due to its constitutive activation, PDAC exhibited high metastatic potential and chemoresistance. Many of the pathway molecules are altered by sialylation^39,40^. The inhibition of PI3K–Akt pathway sometimes is responsible for the activation of Fas-mediated apoptosis in gastric, colon, and prostate cancer^41–43^. Alterations of sialylation of the PI3K pathway molecules cause inactivation of oncogenes like PI3K/mTOR resulting in regression of cancer. Hence, Neu2 may play an important role in desialylation of such pathway molecules which is not fully understood.

Initially, we observed lower expression of Neu2 in different PDAC cell lines and in patient tissues. Moreover, such reduced Neu2 exhibited a significant strong association with clinicopathological characteristics of these patients (Table 1). Accordingly, we aim to establish the role of this cytosolic sialidase on sialylated Fas in Fas-mediated apoptosis in PDAC cell lines with different mutations (Table 2).

**Table 1:** Association of Neu2 expression with clinicopathological characteristics in patients with pancreatic cancer

Gender			
Male			12
Female			8
Age			40–85
Location of tumor			
Head, body			20
T category			
T1			1
T2			11
T3			8
N category			
N0			11
N1			9
M category			
M0			20
M1			0
Surgical procedure			
Pancreatoduodenectomy			20
Histological differentiation			
Well			3
Moderately			16
Poorly			1
Chemotherapy			0
Adjuvant therapy			0
Neu2 expression			
Neu2 negative			17
Neu2 positive			3

**Table 2:** Mutations in pancreatic ductal adenocarcinoma (PDAC) cells

Cell Line	K-ras	AKT2 19q	p16 HD	p16 Mut	13q LOH	BRCA2 Mut	17p LOH	p53 Mut	MKK4 HD
MiaPaCa2	12 Cys	—	HD	—	—	—	LOH	248 Trp	—
AsPc1	12 Asp	Ampl.	—	2 bp del.	LOH	—	LOH	1 bp del.	HD
BxPc3	—	Poss. Ampl.	HD		LOH	—	LOH	220 Cys	—
Panc1	12 Asp	Ampl.	HD		LOH	—	LOH	273 His	—

MIAPaCa2 is derived from primary tumor, having K-RAS activating mutation, p53 inactivating mutation, and p16 homozygous deletion

AsPC1 is derived from ascites having K-RAS activating mutation, p53 inactivating mutation, p16 frame shift mutation, Akt amplification, and MKK4 homozygous deletion

BxPC3 is derived from primary tumor, having Akt amplification, p53 inactivating mutation, and p16 homozygous deletion

PANC1 is derived from primary tumor, having K-RAS activating mutation, p53 inactivating mutation, and p16 homozygous deletion

Here, we demonstrated that overexpression of cytosolic Neu2 increased enzyme activity on the membrane. Enhanced membrane-bound Neu2 exhibited an increased association with α2,6-linked SAs on Fas causing its desialylation and activation as corroborated by decreased association of Fas with an SA-binding lectin, *Sambucus nigra* agglutinin (SNA). Such desialylated Fas guided these cells toward enhanced apoptosis through extrinsic pathway. Additionally, enhanced cytosolic Neu2 desialylated several signaling molecules present in PI3K–Akt/mTOR pathway. All these events accelerated apoptosis by inhibiting this pathway which also causes upregulation of Fas expression and activation. These entire processes diminished the survival of Neu2-transfected drug-resistant PDAC cells through abridged cell migration and invasiveness.

To the best of our knowledge, this is the first information for the presence of Neu2 on the membrane and establishing a link between the function of cytosolic Neu2 for desialylation of membrane-bound Fas. Neu2, therefore, may be a pivotal upstream molecule in regulating apoptosis.

## Results

### **N**eu2 is downregulated in human pancreatic cancer tissues

**Fig. 1:**
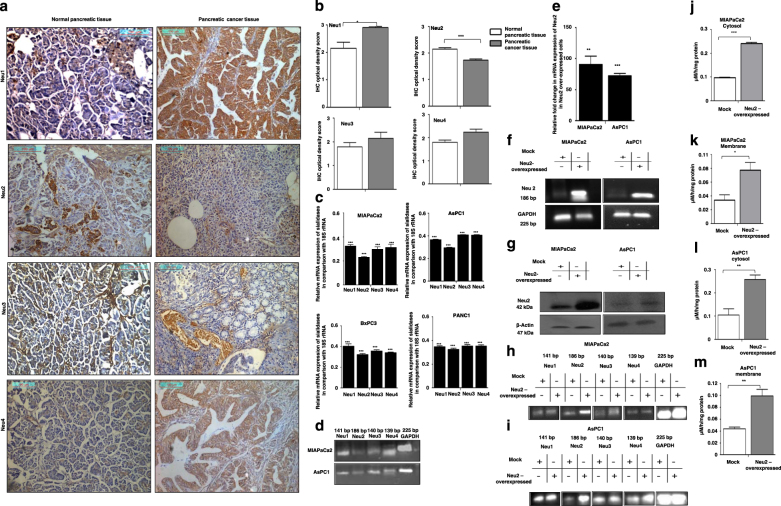
Neu2 is downregulated among the other mammalian sialidases in human pancreatic carcinomas. **a** Expression of four different sialidases in patient’s tissue compared to normal. Tissue samples from pancreatic tumor and their normal counterpart were collected by our clinical collaborator at the Institute of Postgraduate Medical Education and Research Hospital, Kolkata. The Neu1, Neu2, Neu3, and Neu4 protein levels were detected in human pancreatic cancer and normal tissue specimens by immunohistochemistry using respective antibodies. Representative images of pancreatic adenocarcinoma were taken with ×20 magnification, showing high positivity for Neu1, Neu3, and Neu4 than normal tissue, poorly differentiated adenocarcinoma showing reduced expression of Neu2 than normal tissue. **b** Bar graphs represent IHC optical density scores of normal and patients tissue samples for Neu1, Neu2, Neu3, and Neu4 sialidases as measured by ImageJ software. **c** Genetic expression of four different sialidases in MIAPaCa2, AsPC1, BxPC3, and PANC1 cells. RNA was isolated from MIAPaCa2, AsPC1, BxPC3, and PANC1 cell lines. cDNA was prepared by ImPromII-Reverse transcription system according to manufacturer’s protocol. Relative mRNA expression of Neu1, Neu2, Neu3, and Neu4 was evaluated by real-time PCR analysis with specific primers of Neu1, Neu2, Neu3, and Neu4 as described in Table 4. Values are normalized against 18S rRNA expression (*n* = 3 experiments). **d** Genetic expressions of different sialidases were additionally measured in MIAPaCa2 and AsPC1 cell lines by semi-quantitative RT-PCR. The image was visualized and photographed in Bio-Rad Gel Documentation system. GAPDH was used as loading control. **e**, **f** Enhanced Neu2 expression in Neu2-transfected MIAPaCa2 and AsPC1 cells. MIAPaCa2 and AsPC1 cells were transfected with the mock and PcDNA3.1-Neu2 expression vector. Neu2 expression was measured after transfection. Real time and semi-quantitative RT-PCR analysis were done from total RNA extracted from mock and Neu2-overexpressed cells as described in Material and methods to detect the Neu2 expression with the specific primer. Fold change in mRNA expression of Neu2 by real-time PCR analysis in MIAPaCa2 and AsPC1 relative to that of mock-transfected cells were determined. Values were normalized against 18S rRNA expression (*n* = 3 experiments). GAPDH was used as loading control for semi-quantitative RT-PCR. The images were visualized and photographed as described above. **g** Higher level of Neu2 protein in Neu2-transfected MIAPaCa2 and AsPC1 cells. Cell lysate from mock and Neu2-overexpressed PDAC cells was used for western blot analysis to determine the status of Neu2 protein. Blots were probed with the antibody against Neu2. β-actin was used as loading control. **h** Genetic expression of different sialidases by RT-PCR in MIAPaCa2 cells after Neu2 overexpression. GAPDH was used as loading control. **i** Genetic expression of different sialidases by RT-PCR in AsPC1 cells after Neu2 overexpression. GAPDH was used as loading control. **j** Enhanced sialidase activity in cytosol of Neu2-overexpressed MIAPaCa2 cells. The enzyme activity of cytosolic protein (50 µg) of Neu2-overexpressed MIAPaCa2 cells was measured at pH 5.5 using MU-Neu5Ac as substrate by fluorimetric method with excitation at 365 nm and emission at 450 nm. Mock-transfected cells were used for comparison. Data were derived from three independent experiments and presented as mean values ± S.D., significance: ***p < 0.001. **k** Membrane fractions exhibited higher sialidase activity Neu2-overexpressed MIAPaCa2 cells. Sialidase activity in 100 µg of protein of the membrane fractions of Neu2-overexpressed MIAPaCa2 cells was determined at pH 5.5 by fluorimetric method. Data were derived from three independent experiments and presented as mean values ± S.D., significance: *p < 0.05. **l** Enhanced sialidase activity in cytosol of Neu2-overexpressed AsPC1 cells. The enzyme activity in cytosolic protein of Neu2-overexpressed AsPC1 cells was measured as described in **j**. Significance: **p = 0.0095. **m** Membrane fractions exhibited higher sialidase activity Neu2-overexpressed AsPC1 cells. Sialidase activity in the membrane fractions of Neu2-overexpressed AsPC1 cells was determined as described in **k**. Significance: **p = 0.0076

Initially, we compared the status of Neu1/Neu2/Neu3/Neu4 in cancer and normal tissue specimens by immunohistochemistry. Optical density score conferred higher Neu1, Neu3, and Neu4 positivity in the tumor tissues (Fig. 1a, b). In contrast, statistically significant low or undetectable expression of Neu2 was observed in all tissues from 20 patients compared to 20 normal counterparts (Table 1). Interestingly, we observed a strong association of reduced expression of Neu2 with clinicopathological characteristics of these patients. This data suggested that the loss of Neu2 possibly helps higher sialylation status in manifestation of this cancer.

Real-time PCR analysis of sialidases in four representatives PDAC cells namely MIAPaCa2, AsPC1, BxPC3, and PANC1 (Table 2) showed reduced Neu2 expression compared to other three sialidases (Fig. 1c). However, MIAPaCa2 and AsPC1 exhibited lowest Neu2 expression amongst them. Interestingly, they also possess different mutations status. Hence, we have selected these two cells for subsequent study. RT-PCR analysis of all these sialidases also showed similar trend in MIAPaCa2 and AsPC1 (Fig. 1d).

Accordingly, Neu2 was transfected in MIAPaCa2 and AsPC1. Neu2-transfected cells exhibited higher mRNA expression of Neu2 than mock both by real time PCR (Fig. 1e) and RT-PCR (Fig. 1f). Western blot analysis also revealed enhanced level of Neu2 protein (Fig. 1g). However, the expression of Neu1, Neu3, and Neu4 remain unchanged in Neu2-overexpressed MIAPaCa2 (Fig. 1h) and AsPC1 (Fig. 1i).

### Sialidase activity in Neu2-transfected cells

We measured the sialidase activity both in cytosolic and membrane fractions of Neu2-transfected cells separately at pH 5.5 toward an artificial substrate MU-NeuAc (Fig. 1j–m). An increased cytosolic enzyme activity was observed in the Neu2-transfected MIAPaCa2 (0.2414 ± 0.0044 µM/h/mg protein) compared to mock-transfected (0.09842 ± 0.00026 µM/h/mg protein) cells (Fig. 1j). Similar trend was found in Neu2-transfected AsPC1 (0.2579 ± 0.019 µM/h/mg protein) compared to mock (0.1048 ± 0.0266 µM/h/mg protein) (Fig. 1l).

We also found increased level of sialidase activity on membrane in Neu2-overexpressed MIAPaCa2 (mock = 0.0340 ± 0.0077 vs. Neu2 = 0.07767 ± 0.0107 µM/h/mg protein) (Fig. 1k). Neu2-transfected AsPC1 also exhibited similar pattern; mock = 0.04372 ± 0.0027 vs. Neu2 = 0.09933 ± 0.0108 µM/h/mg protein (Fig. 1m). Hence it may be concluded that though Neu2 is a cytosolic enzyme, it also exhibited slightly higher sialidase activity in membrane fraction.

### Neu2 overexpression inhibits cell proliferation

**Fig. 2:**
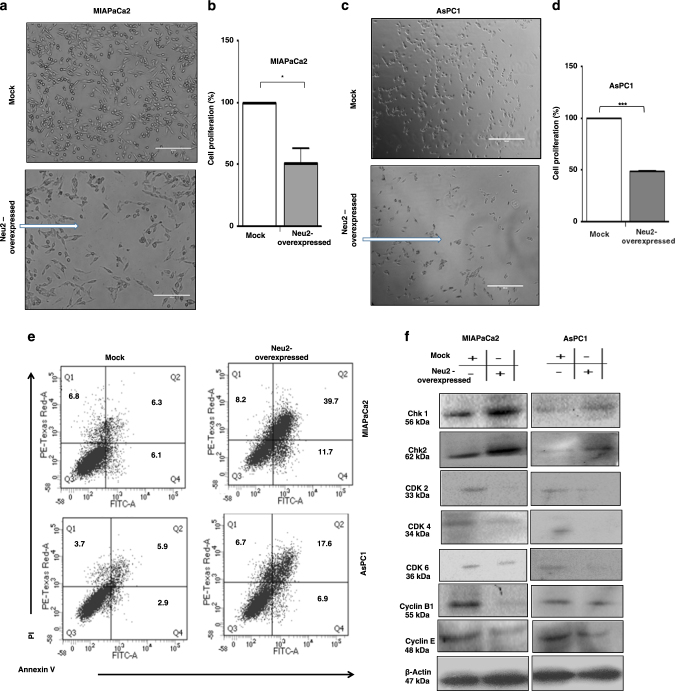
Neu2 overexpression inhibits cell proliferation and modulates cell cycle-related proteins. **a** Neu2-overexpressed MIAPaCa2 exhibited less cell viability. MIAPaCa2 cells were transfected with mock and PcDNA3.1-Neu2 expression vector. These cells were kept in culture medium without serum for 3 days and visualized by a phase contrast microscope (Evos inverted microscope, Life Technologies). The images were obtained with the magnification of ×20. **b** Cell viability of mock and Neu2-overexpressed MIAPaCa2 cells after 3 days of serum starvation which was used as apoptotic stimuli was determined by MTT assay. Cell viability was represented as % proliferation between mock and Neu2-transfected MIAPaCa2 cells. The data are the means ± S.D. of three different experiments. Significance: *p < 0.05. **c** Neu2-overexpressed AsPC1 exhibited less cell viability. Mock and Neu2-overexpressed AsPC1 cells were similarly processed as described in **a**. **d** Cell viability of mock and Neu2-overexpressed AsPC1 cells were processed by MTT assay as shown in **b**. Significance: ***p < 0.0001. **e** Neu2 induces apoptosis in the MIAPaCa2 and AsPC1 cell lines. Effects of Neu2 overexpression on PDAC cells after 24 h of serum starvation was determined using annexinV–PI staining by flow cytometry. The percentage of apoptosis (annexin V/PI) in Neu2-transfected PDAC cells has been shown in the dot plot. Mock-transfected cells were used as controls. **f** Neu2 overexpression modulates cell cycle-related proteins both in MIAPaCa2 and AsPC1 cells. Neu2-induced regulation of known molecular mediators of cell cycle in PDAC cells was analyzed by western blot with the indicated antibodies showing upregulation of Chk1 and Chk2. Downregulation of CDK 2, CDK 4, CDK 6 and cyclin B1 and cyclin E was observed as determined by immunoblot analysis. β-actin was used as loading control

Next, we checked the growth potential of MIAPaCa2 and AsPC1 due to enhanced Neu2 activity by 3-(4,5 dimethyl thiazol-2yl)-2,5 diphenyltetrazolium bromide (MTT) assay. The Neu2-transfected cells revealed an impaired proliferation capacity after 3 days of culture in the serum-starved condition which was used as apoptotic stimuli compared to mock. Less number of viable Neu2-overexpressed MIAPaCa2 (Fig. 2a) and AsPC1 (Fig. 2c) cells compared to mock were observed in microscope.The percent of cell viability was significantly reduced by 51.35 ± 11.74 and 48.67 ± 0.78 in MIAPaCa2 (Fig. 2b) and AsPC1 (Fig. 2d) respectively by MTT.

### Neu2-induced apoptosis is confirmed by annexin V/PI

Since significant cell death was observed in Neu2-transfected cells, we checked the mode of this event after 24 h serum starvation. Neu2 overexpression caused surface phosphatidylserine externalization in MIAPaCa2. AnnexinV positivity was 10.90 ± 0.493 compared to 6.47 ± 0.203 in mock-transfected cells illustrating the features of early apoptosis (Fig. 2e). Neu2-transfected AsPC1 also exhibited higher annexin V positivity.

A substantial number (36.9 ± 1.429) of Neu2-transfected MIAPaCa2 lost membrane integrity and showed late apoptotic bodies as evidenced by both annexin V and PI positivity. AsPC1 also showed a similar trend. Hence it is confirmed that growth inhibition in Neu2-transfected cells was due to apoptosis.

### Neu2 overexpression modulates cell cycle-related proteins

To determine whether growth inhibition induced by Neu2 was associated with the regulation of cell cycle-related proteins, we checked the status of these molecules both in mock and Neu2-transfected cells by immunoblotting (Fig. 2f). Neu2 overexpression upregulated Chk1 and Chk2. Activated Chk1 and Chk2 subsequently led to the reduction of the cyclins and cyclin-dependent kinases. We observed that early G1-cell cycle phase regulatory proteins (CDK4 and CDK6) were downregulated after Neu2 overexpression. Late G1-phase regulatory proteins (cyclin E and CDK2) were also reduced. Additionally, cyclin B1 involved in the transition of G2 to the M phase was decreased. Modulation of all these proteins suggested that enhanced Neu2 hinders cell growth by impeding different checkpoints, hence it may be stated that as an enzyme it affects the overall cell cycle phases.

### Neu2 induces modulation of anti-apoptotic and pro-apoptotic molecules

**Fig. 3:**
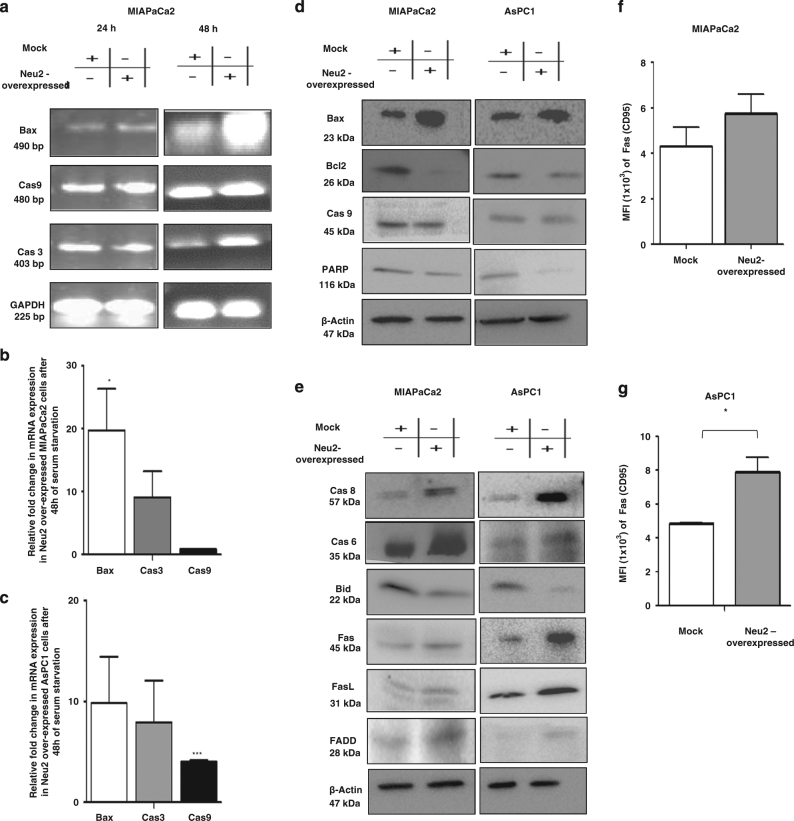
Neu2 induces apoptosis by regulating pro and anti-apoptotic molecules. **a** MIAPaCa2 cells were transfected with mock and PcDNA3.1-Neu2 expression vector and serum starved for 24 and 48 h. RNA was isolated from these cells and cDNA was prepared. RT-PCR analysis of Bax, caspase 9, and casapse3 was done. mRNA expression showed upregulation of pro-apoptotic molecules but no change was found in caspase 9 in Neu2-overexpressed MIAPaCa2 cells compared to mock. GAPDH was used as loading control. The image was visualized and photographed. **b**, **c** Real-time PCR analysis of Bax, caspase 3, and caspase 9 mRNA expressions in MIAPaCa2 and AsPC1 cells after Neu2 overexpression relative to that of mock-transfected cells after 48 h of serum starvation were determined. Values are normalized against 18S rRNA expression (*n* = 3 experiments). **d** Cell lysates were prepared from mock and Neu2-overexpressed MIAPaCa2 and AsPC1 cells and resolved by SDS-PAGE, and then analyzed by western blot with the specified antibodies. Representative immunoblots showing increased Bax, reduced level of both Bcl2 and PARP in Neu2-overexpressed PDAC cells whereas no such change was found in protein level of caspase-9. β-actin was used as loading control. **e** Neu2 induces apoptosis through the extrinsic-mediated apoptotic pathway. Representative immunoblots of Neu2-overexpressed MIAPaCa2 and AsPC1 cells confirmed the reduction of the protein level of Bid and enhanced caspase 8, caspase 6, Fas, FasL, and FADD which are hallmark proteins of the extrinsic apoptotic pathway. β-actin was used as loading control. **f** MIAPaCa2 cells were transfected with mock and PcDNA3.1-Neu2 expression vector then cells were harvested and incubated with PE-Fas (CD95) antibody for analysis by flow cytometry. Neu2 overexpressed in MIAPaCa2 cells showed upregulation of Fas. The data are the means±S.D. of three different experiments. **g** Neu2-overexpressed AsPC1 cells also exhibited enhanced Fas on the cell surface compared to mock-transfected cells. The data are the means±S.D. of three different experiments. Significance: *p < 0.05

We then investigated the probable cause of the observed susceptibility to apoptotic stimuli. We found enhanced mRNA expression of the pro-apoptotic genes (Bax) and effector caspase (caspase 3), but not caspase 9 in Neu2-overexpressed MIAPaCa2 compared to mock-transfected cells after 24 and 48 h (Fig. 3a).

We also observed higher mRNA level expression of Bax and Cas3 in both MIAPaCa2 (Fig. 3b) and AsPC1 (Fig. 3c) by real-time PCR analysis after 48 h. However, the lower genetic expressions of caspase 9 were observed in both the cells.

Western blot analysis also showed higher level of Bax in Neu2-transfected MIAPaCa2 (~6.5 fold) and AsPC1 (~1.5 fold), whereas caspase 9 remained unchanged, which is a hallmark protein for the intrinsic apoptotic pathway (Fig. 3d).

Consistent with these data, Neu2-transfected MIAPaCa2 and AsPC1 exhibited decreased level of anti-apoptotic proteins, Bcl-2 (~15.08 and 2.0 fold) respectively. PARP, a DNA-repairing enzyme also reduced in the Neu2-transfected cells compared to Mock. Overall, the reduction of Bcl-2 and upregulation of Bax supports the notion of involvement of enhanced Neu2 in the apoptosis-regulating pathways in these transfected cells.

### Neu2 induces reduced cell proliferation through extrinsic apoptotic pathway

Interestingly, we observed a significant increase of activated caspase 6 and caspase 8 which are hallmark proteins for extrinsic apoptosis pathway in Neu2-overexpressed MIAPaCa2 and AsPC1 (Fig. 3e). The cross-talk between the cell surface-mediated apoptotic signal and the mitochondria is caused by Bid, and hence its activation is very important. We observed cleavage of Bid along with the enhanced level of FADD. Taken together, these results suggest that caspase-8 and FADD activation along with cleaved Bid in Neu2-transfected PDAC may be induced by activation of the death receptor-mediated apoptosis.

### Enhanced expression of Fas and FasL in Neu2-transfected cells

Next, we searched for the death receptor mechanism in Neu2-transfected cells. We observed a 1.4 and 1.6-fold upregulation of Fas/CD95 in Neu2-transfected MIAPaCa2 (Fig. 3f) and AsPC1 (Fig. 3g) respectively after 24 h by flow cytometry.

Western blot analysis also suggested a significantly enhanced level of Fas and its ligand (FasL) in the transfected cells (Fig. 3e). Therefore, it may be envisioned that though Neu2 is a cytosolic enzyme still it can stimulate apoptosis by the extrinsic pathway involving membrane-bound Fas.

### Neu2 reduces linkage-specific sialylation on cell surface

**Fig. 4:**
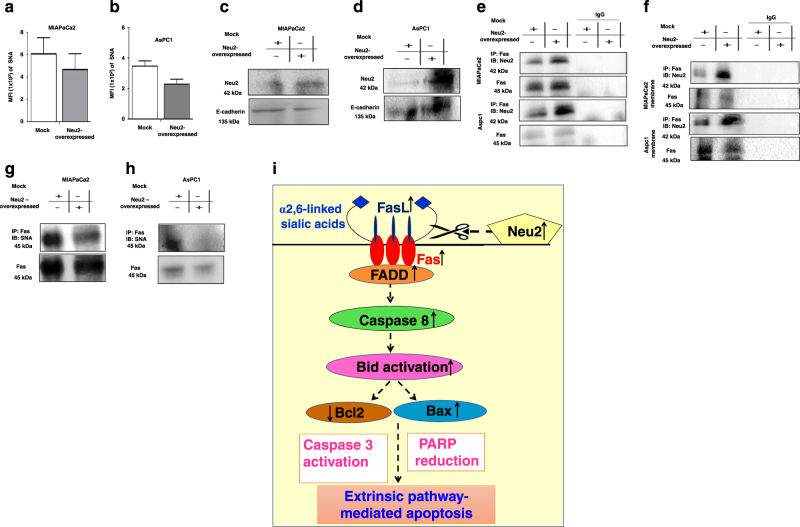
Membrane-bound Neu2 enhances desialylation of α2,6-linked sialic acids on membrane. **a**, **b** Neu2-transfected MIAPaCa2 and AsPC1 cells exhibited decreased α2,6-linked sialic acids. Status of sialic acids on the cell surface was demonstrated through the binding of FITC-conjugated two sialic acid-binding lectins (SNA and MALII). Mock and Neu2-transfected (1 × 10^5^) MIAPaCa2 and AsPC1 cells were washed and resuspended in lectin binding buffer. Cells were incubated separately with FITC-conjugated SNA for 1 h. FITC positivity was acquired by FACS. Data are represented as mean ± S.D. from three independent experiments. **c**, **d** Elevated level of Neu2 expression in membrane fraction in Neu2-overexpressed MIAPaCa2 and AsPC1 cells. Cell lysate was prepared from Neu2-transfected cells; membrane fraction was isolated by ultracentrifugation. Representative immunoblots experiment confirmed increased Neu2 expression in membrane fraction of transfected PDAC cells than mock. E-cadherin was used to show the purity of membrane preparation and also used as loading control. **e** Cell lysate exhibited enhanced association of Fas with Neu2 in Neu2-overexpressed cells. Cell lysates from mock and Neu2-overexpressed MIAPaCa2 and AsPC1 cells were incubated with anti-Fas antibody for overnight and immunoprecipitated. The immuno-complex was resolved by SDS-PAGE. Subsequently, it was identified by anti-Neu2 antibody and detected by ECL. Representative immunoblots of co-immunoprecipitation experiments of cell lysate in PDAC confirmed increased binding of Fas with Neu2 in Neu2-overexpressed cells. IgG was used as negative control. **f** Enhanced association of membrane-bound Neu2 with Fas in Neu2-overexpressed cells. Co-immunoprecipitation experiments with membrane fractions of mock and Neu2-overexpressed MIAPaCa2 and AsPC1 cells confirmed enhanced association of Neu2 with Fas. IgG was used as negative control. **g**, **h** Decreased sialylation of Fas in Neu2-transfected cells. Cell lysate from mock and Neu2-overexpressed MIAPaCa2 and AsPC1 cells were incubated with anti-Fas antibody for overnight and immunoprecipitated. Immuno-complex was resolved, and subsequently incubated with biotinylated-α2,6-linked sialic acid binding lectin (SNA) then developed by avidin-HRP antibody and detected by ECL. Lectin affinity study of Neu2-transfected MIAPaCa2 and AsPC1 cells confirmed decreased association of Fas with SNA. **i** Schematic representation of Neu2-mediated regulation for desialylation of Fas and downstream signaling for inducing extrinsic-mediated apoptosis by activated Fas

So far we have demonstrated presence of Neu2 on plasma membrane (Fig. 1k, m); accordingly, we checked whether it can reduce linkage-specific sialylation on the cell surface of Neu2-transfected MIAPaCa2 (Fig. 4a) and AsPC1 (Fig. 4b) by flow cytometry. We have found reduced FITC-SNA binding with Neu2-overexpressed cells compared to mock as indicated by lower MFI value being 467.9 ± 142 vs. 607.8 ± 144.6 in MIAPaCa2 and 228.7 ± 32.68 vs. 346.7 ± 34.48 in AsPC1 respectively. In contrast, no detectable change was found when α2,3-linked SA binding lectin (MALII) was used under similar condition (data not shown). This indicates that Neu2 possibly cleaves more α2,6-linked SA from sialoglycoproteins on the cell surface of MIAPaCa2 and AsPC1 as α2,6-linked SA is reported to be the specific substrate for Neu2.

### Plausible association of Neu2 on plasma membrane

As we have observed that Neu2 being a cytosolic enzyme can induce apoptosis by the extrinsic-mediated apoptotic pathway involving Fas, so we monitored whether Neu2 is also present on the membrane. Earlier, we have an indication of slightly higher enzyme activity on the membrane of Neu2-transfected MIAPaCa2 (Fig. 1k) and AsPC1 (Fig. 1m) along with less sialylation (Fig. 4a, b).

Now the membrane fractions of these cells were immunoblotted with an anti-Neu2 antibody. We found that higher expression of Neu2 in the membrane of the Neu2 overexpressed MIAPaCa2 (Fig. 4c) and AsPC1 (Fig. 4d). E-cadherin was used to show the purification of membrane fraction. Hence, it may be stated that Neu2 is available on the membrane by an unknown mechanism.

### Neu2 is associated with Fas death receptor

So far we have established the involvement of Fas in mediating apoptosis in Neu2-overexpressed cells through extrinsic pathway. Now, we addressed an obvious question what is the relation between membrane-bound Neu2 and Fas. A co-immunoprecipitation with cell lysate (Fig. 4e) and membrane fractions (Fig. 4f) of Neu2-overexpressed MIAPaCa2 and AsPC1 showed that Neu2 is associated with Fas. Higher expression of membrane-bound Neu2 showed more association with the death receptor suggesting possible enhanced desialylation of Fas.

### Neu2 causes desialylation of α2,6-linked SAs on Fas

Fas is known to have α2,6-linked SAs. Therefore, we checked if the Fas is a direct target for desialylation of α2,6-linked SAs by Neu2. A co-immunoprecipitation with cell lysate of Neu2-overexpressed MIAPaCa2 (Fig. 4g) and AsPC1 (Fig. 4h) with anti-Fas antibody and detected by SNA exhibited decreased α2,6-linked SAs on Fas. The band corresponding to Fas was less intense indicating that Neu2 overexpression causes its de-sialylation. This showed that α2,6-linked SAs are also present on Fas in PDAC and further reconfirms that they are the true substrate for Neu2.

### Neu2 mediates alterations in the sialoglycoprotein profile

To identify a possible molecular link between Neu2 overexpression and its association with Fas, we examined the status of sialoglycoproteins on membrane and cytosolic proteins of transfected cells using SNA and MALII lectins.

**Fig. 5:**
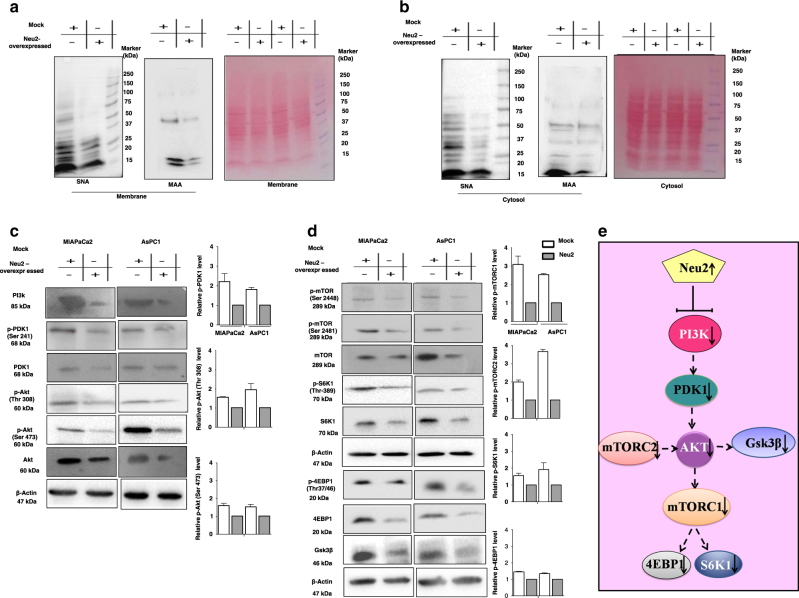
Enhanced Neu2 modulates alterations of sialoglycoproteins which regulates the activity of signaling molecules involved in PI3K–Akt/mTOR. **a**, **b** Enhanced reduction of α2,6-linked sialioglycoproteins in membrane and cytoslic fraction of Neu2-overexpressed cells. Status of sialoglycoproteins profiles present in the membrane and cytosolic fractions purified from mock and Neu2-overexpressed MIAPaCa2 cells were assessed by lectin blotting employing both α2,6- and α2,3-linked sialic acid binding lectins namely SNA and MALII. Ponceau S stained blot was used to indicate equal loading. **c**, **d** The cell lysate was prepared from mock and Neu2-overexpressed MIAPaCa2 and AsPC1 cells and resolved by SDS-PAGE, and then analyzed by western blot with the specified antibodies. Representative immunoblots showed reduction of PI3K–mTOR pathway proteins and its downstream molecules upon Neu2 overexpression in PDAC cells. **e** Pictorial representation of the summary of this pathway modulated by Neu2 overexpression in PDAC cells.

SNA-binding membrane proteins showed significant differences between Neu2-transfected MIAPaCa2 compared to mock-transfected cells (Fig. 5a). The major pattern changes occurred between 20 and 75 kDa proteins which underwent a marked loss of SA indicating these sialoglycoproteins as the possible targets for Neu2. Ponceau S stained blot was used to indicate equal loading. Conversely, the appearance of Neu2 transfection in MIAPaCa2 caused only slight changes in the pattern of α2,3-linked sialoglycoproteins.

Similar changes in the reduction of 20 and 100 kDa sialoglycoproteins profiles were observed in cytosolic proteins of transfected MIAPaCa2 (Fig. 5b). It may be noted that most of the molecules in the PI3K-signaling pathway are between 15 and 100 kDa and they are modulated by their sialylation status. Therefore, enhanced cytosolic Neu2 possibly has a direct role in regulating some signaling pathway by desialylation. Therefore, our observation may provide a possible link between Neu2 activity and alterations of growth factor-mediated signaling due to desialylation by Neu2 overexpression.

### Neu2 impairs the activity of several signaling molecules involved in PI3K–Akt/mTOR pathway

PI3K is known to play a key role in Akt–mTOR signaling and help cancer cells to survive. So far we have demonstrated the association of α2,6-linked sialylated Fas with membrane-bound Neu2 and its activation through desialylation (Fig. 4g, h). Additionally, enhanced cytosolic Neu2 may have a direct effect on regulating PI3K pathway probably by modulation of sialylation of several molecules involved in this pathway. Inhibition of PI3K pathway causes upregulation of Fas which had been reported^42^. However, cross talk between enhanced Neu2, inhibition of the PI3K pathway, and Fas activation has not been explored.

Therefore, we turned our attention to the upstream proteins of this pathway involved in uncontrolled proliferation. We observed a decreased level of PI3K in Neu2-transfected MIAPaCa2 and AsPC1 (Fig. 5c). Neu2 overexpression inhibited PDK1 phosphorylation at Ser241. Thus this sialidase possibly reduces the PDK1-mediated Akt/mTOR signaling. PDK1 partially activates Akt phosphorylation at Thr308, we have also found decreased phosphorylation in Neu2-overexpressed cells.

Furthermore, to check the effect in the intracellular phases of signaling, we evaluated specific phosphorylation level of mTOR complexes and its downstream signaling proteins in Neu2-transfected MIAPaCa2 and AsPC1 (Fig. 5d). They exhibited reduced phosphorylation at Ser2481 indicating mTORC2-specific phosphorylation. We examined the phosphorylation status of AKT at Ser473, which is a selective substrate of mTORC2 for confirming this upstream inhibition of mTORC2 signaling in these Neu2-transfected cells. We observed reduced expression of phospho-AKT at Ser473 (Fig. 5c). This observation strongly suggested that Neu2 overexpression had a significant role in the reduction of mTORC2 activity, which in turn decreased the phosphorylation of its substrate AKT at the Ser473.

We also detected slightly reduced levels of phosphorylation of active mTORC1 at Ser 2448 in Neu2-transfected MIAPaCa2 and AsPC1 (Fig. 5d). As PDK1 pathway regulates mTORC1 activity which consequently controls the downstream signaling molecules, therefore, we studied the phosphorylation of S6K1 and 4E-BP1. We observed reduced phosphorylation of S6K1 at Thr389, 4E-BP1 at Thr37/46, and GSK3β after overexpression of Neu2 in these cells suggesting the involvement of mTORC1 also. Taken together, all these observations suggest that overexpression of Neu2 causes reduction of growth factor-mediated several signaling molecules in the PI3K pathway, as a whole through modulation of sialylation which also could activate Fas. Thus, the activated Fas possibly deregulates the overall pathways for cell proliferation.

### Enhanced Neu2 reduces cell migration and invasion through the modulation of VEGF, VEGFR, and MMP9

**Fig. 6:**
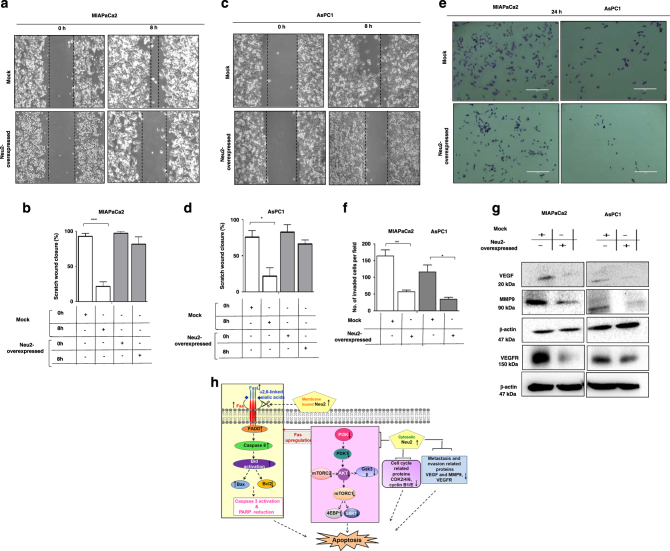
Neu2 overexpression reduces migration and invasion, by controlling angiogenic and metastasis related proteins. **a**-**d** Inhibition of migration in Neu2-transfected MIAPaCa2 and AsPC1 cells. **a** MIAPaCa2 cells were transfected with mock and Neu2 plasmid in a six-well plate. In every well, a scratch was made by 2.5 µl tips. Representing images showed the filling of gaps in each well after 8 h in mock cells compared to Neu2-transfected cells. **b** Area of closure was calculated and graphically represented. **c** AsPC1 cells were transfected and processed similarly. Representing images showed minimal filling of gaps in each well in transfected cells. **d** Area of closures was shown. Data are represented as mean ± S.D. from three independent experiments. **e** MIAPaCa2 and AsPC1 cells exhibited lower invasiveness when Neu2 was overexpressed. Inverted light microscopic images of these cells showed decreased invasiveness due to Neu2 transfection. Mock-transfected cells were used as control. **f** Three randomly selected fields were counted represented graphically. Data are represented as mean ± S.D. from three independent experiments. **g** Neu2-overexpressed MIAPaCa2 and AsPC1 cells exhibited decreased level of VEGF, VEGFR, and MMP9. Representative immunoblots showed decreased proteins level of metastasis and invasiveness related proteins (VEGF, VEGFR, and MMP9) in Neu2-overexpressed PDAC cells. β-actin was used as loading control. **h** Pictorial representation of the summary of this study

Next, we checked the role of enhanced Neu2 in metastasis through migration and invasion assays. We observed there was no significant change in cell migration of Neu2-overexpressed MIAPaCa2 (Fig. 6a, b) and AsPC1 (Fig. 6c, d) after 8 h by scratch wound assay (Table 3) compared to mock.

**Table 3:** Values of migration and invasion assay in MIAPaCa2 and AsPC1 cells

Migration assay	MIAPaCa2 scratch wound closure (%)		AsPC1 scratch wound closure (%)	
	0 h	8 h	0 h	8 h
Mock	93.30 ± 3.9	22.22 ± 5.9	75.92 ± 9.26	22.17 ± 11.5
Neu2	97.77 ± 2	82.22 ± 9.7	83.32 ± 9.62	66.57 ± 5.56
Invasion assay	: Number of invaded cells per field			
Mock	24 h	24 h		
	163.7 ± 18.35	116.0 ± 20.74		
Neu2	57.0 ± 5.13	34.67 ± 5.55		

Additionally, we investigated the potential ability for invasiveness of Neu2-overexpressed cells. Mock-transfected MIAPaCa2 and AsPC1 invade Matrigel layer after 24 h (Fig. 6e). However, not much invasion of Neu2-transfected cells was observed. The number of invaded cells per-field in mock was much higher than Neu2-transfected cells (Fig. 6f). Taken together, Neu2-transfected cells possess less invasive property (Table 3).

To further strengthen the reduced inability of Neu2-transfected cells in metastasis and invasion, we have checked the status of a few specific hallmark proteins like vascular endothelial growth factor (VEGF), vascular endothelial growth factor receptor proteins (VEGFR), and matrix metalloproteinase 9 (Fig. 6g). All these molecules were decreased compared to mock-transfected cells. Hence, it seems that not only enhanced Neu2 induces apoptosis but it can also have the potential to reduce the metastasis even in drug-resistant MIAPaCa2.

## Discussion

Aberrant sialylation in signaling pathways involved in altering their functions leads to abnormal cellular signaling causing major changes in cellular behavior to induce resistance to apoptosis and invasiveness^44^. Sialyltransferases and sialidases are two important players in this event. The key achievement of our study is to provide evidence for the enhancement of cytosolic Neu2 on the membrane in the Neu2-overexpressed PDAC cells. We have demonstrated for the first time a close association of membrane-bound Neu2 with sialylated Fas, which plays a major regulatory upstream molecule for the activation of Fas through desialylation. Activated Fas, after desialylation, enhanced several apoptotic molecules through extrinsic apoptotic pathway caused by increased Neu2 on the membrane. Fas activation is also modulated by desialylation of several molecules involved in the PI3K pathway by enhanced cytosolic-Neu2 activity. The effect of all these was reflected in decreased metastasis, invasiveness, and cellular proliferation due to decreased PI3K-pathway activity through modulation of sialylation which helps in upregulation of Fas activation due to enhanced Neu2. Moreover, a significant strong association of lower expression status of Neu2 with clinicopathological characteristics in patient tissues has also been demonstrated.

Several reports suggested that higher sialylation status causes cell motility, adhesion, and metastasis by modulating α2,3 sialyltransferase, ST3GalIII, galactosyl transferases in PDAC^25^. ST6Gal1 also plays an important role in regulating the invasiveness in a fructose-responsive manner^24^. This enzyme enhances α2,6-sialylation of Fas which confers protection against Fas-mediated apoptosis and facilitates tumor progression in colon cancer^36^. We have also shown α2,6-sialylation of Fas in PDAC. To the best of our knowledge, this is the first report on Fas-sialylation in PDAC.

Conformation changes of glycoproteins, recognition of functional molecules especially in relation to cancer progression are usually caused by removal of SAs which was catalyzed by a sialidase^26^. These observations comprised the preface of our study to evaluate the effects of cytosolic-sialidase Neu2, which is present in very low amount in PDAC cells. The role of Neu2 in differentiation events is an extremely regulated process^29^. Enhanced Neu2 expression leads leukemic cells towards apoptosis^31^. However, several tumor cells generally with a high degree of malignancy are lacking this sialidase^45^.

Due to lack of adequate understanding of the mechanism by which Neu2 regulates tumor cell behavior in pancreatic carcinoma, we aimed to explain the significance of Neu2 downregulation. Accordingly, we overexpressed Neu2 in drug-resistant PDAC cells.

Neu2 overexpression showed apoptosis susceptibility toward apoptotic stimuli such as serum deprivation which significantly reduced the proliferation rate. More importantly, this event pushed Neu2-overexpressed cells toward apoptosis as revealed by an increased number of cells in the late apoptotic stage by affecting important cell cycle regulatory molecules along with increasing the expression of the pro-apoptotic and decreasing the anti-apoptotic proteins. Interestingly, caspase 6 and 8 were activated but not caspase 9 hinting for activation of the extrinsic pathway. Furthermore, we observed higher enzyme activity in the membrane fraction. This outcome gave us first notion that Neu2 probably present in the membrane in transfected cells by an unknown mechanism which desialylated α2,6-linked SAs of cell surface sialoglycoproteins. Interestingly, α2,6-linked SAs are considered to be the main substrate for Neu2 in pancreatic cancer. This was corroborated by the significant differences in sialylation profile of various sialoglycoproteins both in membrane and cytosol between mock and Neu2-transfected cells suggesting desialylation may be the cause of the effects which was activated by Neu2. Changes were more prominent in cleaving α2,6-linked sialoglycoproteins. No appreciable changes in the level of α2,3-linked SAs were observed.

The activation of the Fas-apoptotic pathway by the inhibition of the PI3K/Akt pathway was reported in colon cancer^42^. It was also found that inhibition of PI3K pathway causes Fas activation in gastric cancer and in prostate cancer^41,43^. Thus, considerable efforts have been made for understanding the molecular crosstalk between PI3K and Fas-mediated cell death in PDAC cells. All these events have been demonstrated pictorially in Fig. 6h.

Taken together, to the best of our knowledge, this is the first evidence demonstrating cytosolic Neu2 on the membrane which plays as an upstream important molecule for establishing a cross-talk between Neu2, activation of Fas, induction of extrinsic pathway and inhibition of PI3K pathway to induce apoptosis in drug-resistant human pancreatic cancer cells.

## Material and methods

### Chemicals

2′-(4-Methylumbelliferyl)-α-d-*N*-acetylneuraminic acid (4-MU-NeuAc), 4-methylumbelliferone (MU) and MTT were from Sigma (St. Louis, USA). Anti-Neu1/Neu3 (Invitrogen)/Neu2 (Abnova, Taiwan)/Neu4 (Pierce), PE-Fas (CD95, BD Bioscience, USA) antibodies and all other antibodies were from Cell Signalling Technology (Danvers, USA). PcDNA3.1-Neu2 was the kind gift from Dr. Eugenio Monti.

### Patient samples

The surgical specimens were obtained from pancreatic cancer patients who underwent resection of their tumors from Institute of Postgraduate Medical Education and Research Hospital, Kolkata. The Institutional Human Ethical Committee had approved the study. Consent of the patients or their parents/guardians were taken for the sample needed for this study. All the clinicopathological details of patients included in this study are summarized in Table 1.

### Immunohistochemistry

For immunohistological staining, pancreatic tumors and adjacentnormal tissues were fixed, embedded in paraffin and microtome-sectioned. To assess the expression of all four sialidases (Neu1/Neu2/Neu3/Neu4), the sections were heated in 0.01 M citrate buffer (pH 6.0) for recovery of antigen and incubated with the respective antibodies. The tissue samples were photographed at ×20 magnification and IHC optical density score was measured by ImageJ software. All 40 samples were used for IHC of Neu2, whereas 10 samples were evaluated for Neu1, Neu3, and Neu4.

### Cell culture

MIAPaCa2, AsPC1, BxPC3, and PANC1 were purchased from ATCC (Table 2). MIAPaCa2, AsPC1, and BxPC3 cells were cultured in RPMI-1640 medium, and PANC1 was cultured in IMDM medium supplemented with heat-inactivated FCS [10% (v/v)], l-glutamine (0.002 M), antibiotics, and antimycotics (Medium A). STR profiling of MIAPaCa2 and AsPC1 was done at University of Nebraska Medical Center, USA.

### Detection of genetic expression of sialidases

Total RNA from cells was extracted using RNeasy mini kit following the manufacturer’s instruction. First strand cDNA was synthesized by ImPromII-Reverse transcription system according to manufacturer’s protocol.

Semi-quantitative RT-PCR was performed on PTC-100 (MJ Research, GMI Ramsey, MN) using PCR kit with specific primers (Table 4). Expression of GAPDH was used as a housekeeping gene. Real-time PCR was performed using a DyNAmo Flash SYBR Green qPCR Kit. Relative amounts of target mRNA were quantitated using the LightCycler 96 (Roche) software with 18S rRNA as internal control. Neu2 level was also detected by western blot analysis using anti-Neu2 antibody.

**Table 4:** List of primers

	Forward primer	Reverse primer
Neu1	5′-CCTGGATATTGGCACTGAA-3′	5′-CATCGCTGAGGAGACAGAAG-3′
Neu2	5′-AGAAGGATGAGCACGCAGA-3′	5′-GGATGGCAATGAAGAAGAGG-3′
Neu3	5′-TGAGGATTGGGCAGTTGG-3′	5′-CCCGCACACAGATGAAGAA-3′
Neu4	5′-TCACTCCTTCGCCTTCTA-3′	5′-GGCATTGCAGTAGAGGAA-3′
Bax	5′-GGGGACGAACTGGACAGTAA-3′	5′-CCTCCCAGAAAAATGCCATA-3′
Cas3	5′-TGGAATTGATGCGTGATGTT-3′	5′-TCAAGCTTGTCGGCATACTG-3′
Cas9	5′-GCTTAGGGTCGCTAATGCTG-3′	5′-GTGCTGAACATCCCACAATG-3′
GAPDH	5′-GGAAGGACTCATGACCACAG-3′	5′-GTCAGGTCCACCACTGACAC-3′

### Transfection of PcDNA3.1-Neu2 into pancreatic cancer cells

MIAPaCa2 and AsPC1 cells (1 × 10^6^/well) in Medium A at 80% confluence were transfected with Neu2 cDNA inserted into the pcDNA3.1 expression vector by the transfection reagent, lipofectamine-ltx according to the manufacturer’s instructions in a six-well plate. The transfection mixture was removed next day and replaced with fresh medium. Mock transfection was used as the control in all the experiments.

### Sialidase assay

Neu2-transfected cells were cultured for 48 h, resuspended in phosphate-buffered saline (PBS), containing pepstatin A (1.0 μg/ml), aprotinin (10 μg/ml), and leupeptin (10 μg/ml). The cells were lysed by sonication (Qsonica-LLC, XL-2000 series, Newtown, CT, USA) and centrifuged at 800 ×* g* for 10 min. The supernatant was further centrifuged at 1,000,00 ×* g* for 30 min to separate cytosolic and membrane fractions. The sialidase activity in cytosol (50 μg) and membrane (100 μg) was determined by using a fluorimetric assay with 4-MU-Neu5Ac (400 μM) as a substrate. The reaction mixture was incubated at 37 °C for 1 h in sodium acetate buffer (50 mM, pH 5.5). Enzymatic activity was expressed as µM of product per h/mg protein.

### Cell viability assay

Mock and Neu2-transfected cells (1 × 10^4^/100 μl) were seeded in 96-well culture plates under serum-depletion conditions for 72 h and subsequently incubated with MTT (100 µg/ml) for 3 h. Formazan crystals were dissolved in DMSO and OD was taken at 550 nm in an ELISA reader (Thermo Scientific, USA). In parallel, the viability of the cells was also monitored by inverted microscopy and the microscopic images were captured after 72 h.

### Annexin V–PI positivity

Mock and Neu2-transfected cells (1 × 10^6^) were cultured in absence of serum for 24 h. Cells were suspended in annexin V-binding buffer incubated for 45 min in dark at 25 °C. AnnexinV and PI were added according to the manufacturer’s instruction and incubated for 20 min in dark at 4 °C. Acquisition of annexin V and PI positive cells were analyzed by flow cytometry (FACS) (BD LSRFORTESSA) using BD FACSDiva 8.0 software.

### Apoptosis detection assays

Mock and Neu2-transfected cells (1 × 10^6^) to apoptotic stimuli was evaluated by culturing cells under serum-depletion conditions for 48 h. Different types of caspases and pro-apoptotic molecules both in genes and proteins levels were detected by RT-PCR and qRT-PCR, western blotting using specific primers (Table 4), and respective antibodies.

### Detection of Fas (CD95)

Neu2 and mock-transfected cells (5 × 10^5^) were washed, resuspended in PBS (100 µl), and incubated with PE-conjugated anti-Fas (CD95) antibody for 30 min in the dark and subsequently acquired by FACS.

### Detection of linkage-specific SAs through lectin binding by FACS

Presence of linkage-specific SAs in MIAPaCa2 and AsPC1 cells was explored by using two sialic acid-binding lectins namely SNA and *Maackia amurensis agglutinin* (MALII). Mock and Neu2-transfected (1 × 10^5^) cells were washed and resuspended in lectin binding buffer (20 mM Tris, 0.5 M NaCl, 2.0 mM MnCl_2_, 2.0 mM MgCl_2_, 2.0 mM CaCl_2_). Cells were incubated separately with FITC-conjugated SNA and MALII for 1 h (5.0 μg/ml at 4 °C), and FITC positivity was acquired by FACS.

### Co-immunoprecipitation

For detection of the association of Fas with Neu2, mock and Neu2-transfected PDAC cells were lysed. Total cell lysate protein (200 μg) was incubated with the anti-Fas antibody (1:100) overnight at 4 °C. Immuno-complex was incubated with protein A-Sepharose 4B for 3 h, resolved by SDS-PAGE, and subsequently identified using the anti-Neu2 antibodies. Membrane fraction of mock and Neu2-transfected MIAPaCa2 and AsPC1 cells was similarly processed^46^. Rabbit IgG was used as a negative control.

### Detection of sialoglycoproteins on Fas by co-immunoprecipitation with SNA

To detect the status of α2,6-linked sialic acids on Fas in Neu2-overexpressed MIAPaCa2 and AsPC1, cell lysate was incubated with the anti-Fas antibody (1:100) overnight at 4 °C. Immuno-complex was incubated with protein A-Sepharose 4B for 3 h, resolved by SDS-PAGE, and subsequently detected by using the biotinylated SNA and developed with avidin-HRP. Mock-transfected cells were processed similarly for comparison.

### Glycoprotein analysis

Proteins (100 µg) isolated from the membrane and cytosolic fractions of mock and Neu2-transfected MIAPaCa2 cells were separated on (4–20%) gradient SDS-PAGE and transferred onto a PVDF membrane. α2,6- and α2,3-linked SAs were identified by incubating with biotinylated SNA and MALII separately and subsequently developed with avidin-HRP. Ponceau S stained blot was used to indicate equal loading.

### Migration assay

Mock and Neu2-transfected MIAPaCa2 and AsPC1 cells (1 × 10^6^) were cultured to >80% confluency in a six-well plate. Three separate scratch wounds were made and incubated for 24 h. Number of cells that moved into the scratched area was observed, and images were taken by using phase contrast microscopy (EVOS, Life Technologies).

### Invasion assay

Mock and Neu2-overexpressed MIAPaCa2 and AsPC1 cells (2.5 × 10^4^) were suspended in medium (100 μl) without FBS and added to the upper chamber of the insert (6.5 mm diameter, 8 μm pore size; Becton Dickson). Then, it was placed in a 24-well plate with medium and 10% FBS (900 μl). After 24 h, the invaded cells were fixed with 3.7% formaldehyde and stained with crystal violet solution. Cells on the upper side of the insert were removed by a cotton swab. Three randomly selected fields on the lower side of the insert were photographed, and the migrated cells were counted.

### Statistical analysis

All the data were from three independent experiments and statistical analysis was performed using Graph Pad Prism 5. Two tail Student's *t* test was used to detect the differences between the groups. Standard error bars represent the standard deviation of the mean (±S.D.) and *p < 0.05 represented the significant differences between the means of the two test groups.
