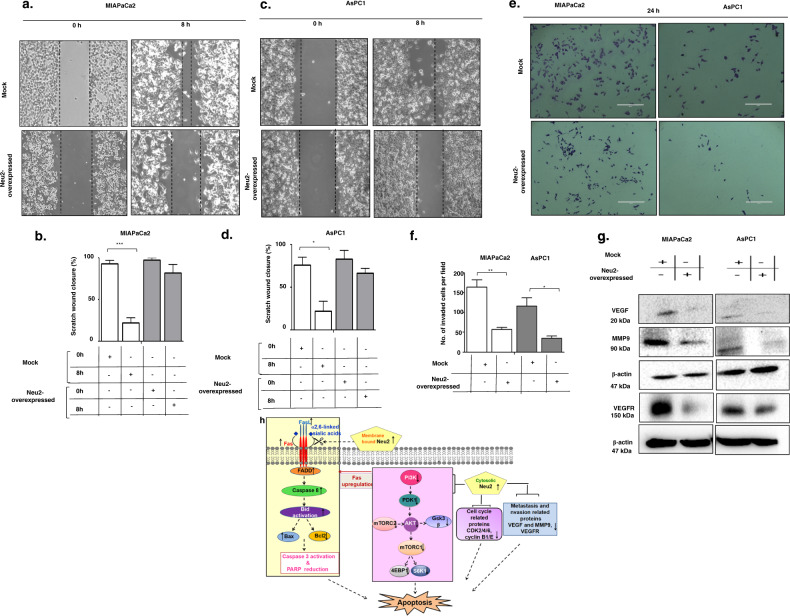# Correction: Association of cytosolic sialidase Neu2 with plasma membrane enhances Fas-mediated apoptosis by impairing PI3K-Akt/mTOR-mediated pathway in pancreatic cancer cells

**DOI:** 10.1038/s41419-022-04714-y

**Published:** 2022-03-16

**Authors:** Shalini Nath, Chhabinath Mandal, Uttara Chatterjee, Chitra Mandal

**Affiliations:** 1 Cancer Biology and Inflammatory Disorder Division, Council of Scientific and Industrial Research (CSIR), Indian Institute of Chemical Biology (IICB), 4, Raja S.C. Mullick Road, Jadavpur, Kolkata, 700032 West Bengal India; 2 National Institute of Pharmaceutical Education and Research, IICB, CSIR, Kolkata, 700032 West Bengal India; 3 Department of Pathology, Institute of Postgraduate Medical Education and Research, Institute of Post-Graduate Medical Education and Research Hospital, Kolkata, 700020 West Bengal India

Correction to: *Cell Death and Disease* 10.1038/s41419-017-0191-4, published online 12 February 2018

The original version of this article unfortunately contained a mistake. In Figure 6a, row 1, column 1, top left box, the control figure for “MIAPaCa2 Mock (0 h)” was incorrect. This error has now been rectified. The correct figure 6a is shown below with the correct control image of MIAPACA2 Mock cells at (0h). The authors would like to apologize for any inconvenience this may have caused.